# Respiratory syncytial virus in pediatric patients with severe acute respiratory infections in Senegal: findings from the 2022 sentinel surveillance season

**DOI:** 10.1038/s41598-023-47015-w

**Published:** 2023-11-21

**Authors:** Mamadou Malado Jallow, Moussa Moise Diagne, Samba Niang Sagne, Fatime Tall, Jean Baptisse Niokhor Diouf, Djibril Boiro, Marie Pedapa Mendy, Ndiendé Koba Ndiaye, Davy Kiori, Sara Sy, Déborah Goudiaby, Cheikh Loucoubar, Gamou Fall, Mamadou Aliou Barry, Ndongo Dia

**Affiliations:** 1 Département de Virologie, Institut Pasteur de Dakar, Dakar, Senegal; 2 Institut Pasteur de Dakar, Unité d’Epidémiologie Des Maladies Infectieuses, 36, Avenue Pasteur, B.P. 220, Dakar, Senegal; 3 Hôpital Des Enfants Albert Royer de Fann, Dakar, Senegal; 4 Hôpital Roi Baudoin de Guediawaye, Dakar, Senegal; 5 Centre Hospitalier Abass Ndao, Dakar, Senegal

**Keywords:** Evolution, Genetics, Microbiology, Molecular biology, Diseases, Medical research

## Abstract

In 2022, many regions around the world experienced a severe respiratory syncytial virus **(**RSV) epidemic with an earlier-than-usual start and increased numbers of paediatric patients in emergency departments. Here we carried out this study to describe the epidemiology and genetic characteristics of RSV infection in patients hospitalized with severe acute respiratory infections in 2022. Samples were tested for RSV by multiplex real time reverse transcription polymerase chain reaction. Subsequently, a subset of RSV positive samples was selected for NGS sequencing. RSV was detected in 16.04%, among which RSV-A was confirmed in 7.5% and RSV-B in 76.7%. RSV infection were more identified in infants aged ≤ 11 months (83.3%) and a shift in the circulation pattern was observed, with highest incidences between September–November. Phylogenetic analyses revealed that all RSV-A strains belonged to GA2.3.5 genotype and all RSV-B strains to GB5.0.5a genotype. Three putative N-glycosylation sites at amino acid positions 103, 135, 237 were predicted among RSV-A strains, while four N-linked glycosylation sites at positions 81, 86, 231 and 294 were identified in RSV-B strains. Globally, our findings reveal an exclusive co-circulation of two genetic lineages of RSV within the pediatric population in Senegal, especially in infants aged ≤ 11 months.

## Introduction

Acute lower respiratory infection (ALRI) caused by respiratory syncytial virus (RSV) has gained recognition as a global health problem with a high burden of disease^1^. Infants and children under 5 years old are particularly susceptible to severe diseases caused by RSV, manifesting as a spectrum of upper and/or lower respiratory tract infections including bronchiolitis and pneumonia^2^. Globally, the virus has led to 33 million episodes of ALRI, 3.6 million hospital admissions, 26,300 in-hospital deaths, and 101,400 RSV-attributable overall deaths in children younger than 5 years^3^. In addition, 99% of RSV-related deaths globally occur in low- and middle-income countries, making such infections a high priority for this setting^4^. Although often characterized as a pediatric disease, RSV infection in adults represents a substantial health burden^5^. Mortality attributable to RSV in adults aged 65 years or older is estimated to be 7.2 per 100,000 person-years, and 8% of RSV ALRI among older adults admitted to hospital was reported to result in death in the USA^1^. RSV is highly seasonal as RSV epidemics tend to occur in the winter in temperate regions^6^ and during the rainy season in tropical countries^7^. Despite the significant public health impact and global economic burden imputed to this pathogen, approved vaccines were recent^8^ with a currently limited access, especially for low income resources countries were RSV immunization program are lacking. Also, a RSV monoclonal antibody product (palivizumab) is available for immunoprophylaxis in a limited high-risk infant population^9^. Therefore, for the time being, measures preventing the spread of RSV remain the most promising means of controlling these seasonal epidemics^10^.

Respiratory syncytial virus (RSV) is a non-segmented negative-sense single-strand ribonucleic acid (RNA) genome of approximately 15.2 kb packaged in a lipid envelope^2,11^. This genome encodes eleven proteins, including attachment (G) and fusion (F) glycoproteins which contain neutralising antibody epitopes capable of inducing a neutralising antibody response, and are targeted in RSV vaccine development strategies^12^. This enveloped virus is a member of the genus *Ortopneumovirus* in the family *Pneumoviridae* and has only one serotype, divided into antigenic subgroups A (RSV-A) and B (RSV-B)^13,14^. Within each subgroup there are numerous genotypes, which have historically been classified based on the gene sequences of the Hypervariable Region 2 (HVR2) of glycoprotein G^15,16^. Usually, both subtypes co-circulate during seasonal epidemic periods with alternating patterns of predominance over time^17^, and currently the most frequent genotypes worldwide are ON1 for RSV-A and BA for RSVB, characterized by duplications of a 72 and a 60 nucleotides in the G gene, respectively^18,19^.

In December 2019, the world experienced the beginning of the coronavirus disease 2019 (COVID-19) pandemic, caused by a novel coronavirus designated as severe acute respiratory syndrome coronavirus 2 (SARS-CoV-2)^20^. The global impact at the public health level was catastrophic, with millions of hospitalizations and deaths^17^. Senegal confirmed its first COVID-19 case on March 2, 2020 when the country had already prepared for its arrival, including rapid detection of cases, patient’s isolation, tracing and quarantine of contacts^21^. Nevertheless, COVID-19 cases increased, and consequently mitigation measures were taken by health authorities, including travel restrictions and border closures, curfews, physical distancing and mandatory wearing of face masks as part of a comprehensive lockdown^22^. These sanitary measures adopted to reduce SARS-CoV-2 transmission, not only impacted COVID-19 but may have also deeply modified the natural course of seasonal viral infections, such as RSV^23,24^. However, with the easing of COVID-19 imposed restrictions, many regions experienced a severe RSV epidemic with an earlier-than-usual start and increased numbers of pediatric patients in emergency departments, partly due to a lack of protective immunity in the community following a lack of exposure from the previous season^10,25,26^. In Senegal, a surveillance of Severe Acute Respiratory Infections (SARI) which falls within the scope of the 4S (Sentinel Syndromic Surveillance in Senegal) network activities was set up in healthcare centers in the capital city Dakar since 2015 with the aim to allow the Ministry of Health to quickly detect and alert any abnormal health event^27^. So here, we carried out a study which describes the epidemiology and genetic characteristics of RSV infection in patients hospitalized with SARI in 2022 after the alleviation of non-pharmaceutical interventions due to the COVID-19 pandemic.

## Results

### RSV confirmed cases

**Figure 1:**
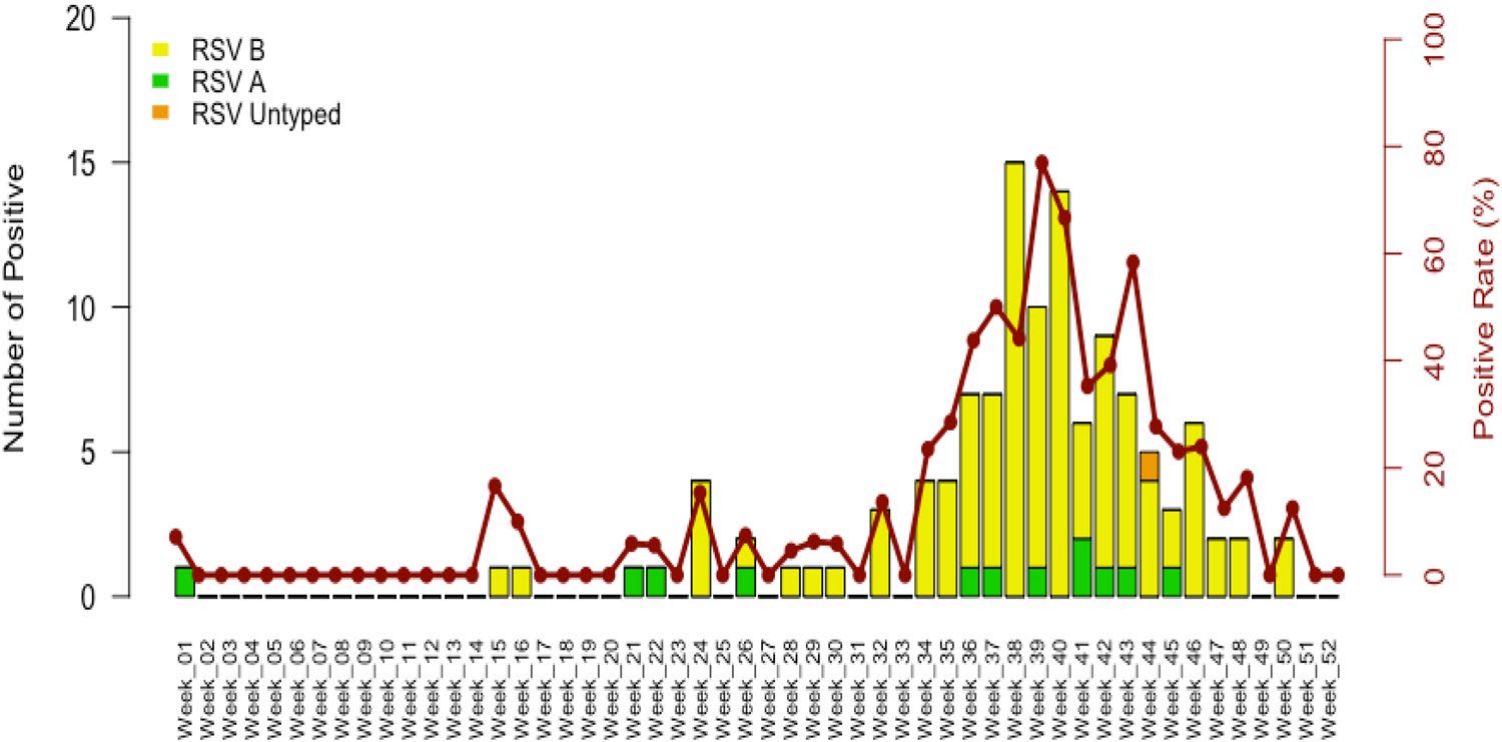
Weekly distribution of RSV among inpatients in Senegal from January to December 2022. Bars represent the proportions of RSV cases for each epidemiological week and the curve represent the positivity rates.

During January, 1 to December 31 2022, a total of 748 SARI samples were received at the National Influenza Center and analyzed by multiplex reverse transcription polymerase chain reaction (RT-PCR). Of these patients, 52.3% (391/748) were male and the median age of the study population was 11 months (interquartile range [IQR]: 4.8 years), with 50.7% (379/748) of all patients being ≤ 11 months old. Overall, RSV was detected in 16% of all samples (120/748), among which RSV type A (RSV-A) was confirmed in 10% (12/120), whereas RSV type B (RSV-B) was encountered in 89.2% (107/120). For the remaining RSV positive sample (0.8%; 1/120), the type could not be determined due to a low viral load. (Table 1). Coinfections of RSV-A and RSV-B were not encountered in any of tested samples. RSV‐B cases outnumbered those of the RSV‐A throughout the study period as showed in Fig. 1. During the first trimester of this study (calendar week 1 to calendar week 14), there was almost no circulation of RSV with only 1 case (RSV-A) in the first calendar week of the year (January). However, we noticed a gradual increase in RSV cases from calendar week 24, reaching a peak in activity between weeks 38 and 40 (September–October) (Fig. 1).

**Table 1:** Demographic characteristics and clinical parameters of RSV-associated SARI patients, Senegal January-December 2022.

RSV positive	Overall	Total tested (n = 748)				Crude OR	Adjusted OR
		RSV A	RSV B	Untyped RSV	p-value		
Characteristics	(n = 120)	(n = 12)	(n = 107)	(n = 1)		(95% CI)	(95% CI)
Gender, n (%)							
Male	55 (45.8)	6 (50.0)	49 (45.8)	0 (0.0)	1	1	1
Female	65 (54.2)	6 (50.0)	58 (54.2)	1 (100)	0.1	0.73 [0.5—1.1]	0.69 [0.45—1.05]
Age group, n (%)							
≤ 11 months	100 (83.3)	11 (91.7)	88 (82.2)	1 (100)	1	1	1
1–2 years	11 (9.2)	1 (8.3)	10 (9.3)	0 (0.0)	5.63 e^−05^	0.3 [0.1—0.5]	0.28 [0.15–0.5]
3–5 years	5 (4.2)	0 (0.0)	5 (4.7)	0 (0.0)	0.001	0.1 [0.03–0.4]	0.15 [0.036–0.4]
6–10 years	2 (1.7)	0 (0,0)	2 (1.9)	0 (0,0)	0.007	0.07 [0.004–0.3]	0.06 [0.003–0.3]
11–15 years	0 (0.0)	0 (0,0)	0 (0.0)	0 (0.0)	0.9	–	–
> 15	1 (0.8)	0 (0,0)	1 (0.9)	0 (0,0)	0.001	0.04 [0.002–0.17]	0.03 [0.002–0.16]
Mean age (95% CI)	0.8 [0.3–1.4]	0.2 [0–0.3]	0.9 [0.3–1.5]	–		–	–
Clinical manifestation, n (%)							
Fever	52 (43.3)	12 (100)	107 (100)	1 (100)	0.0003	0.48 [0.32—0.72]	0.46 [0.31—0.69]
Cough	91 (75.8)	12 (100)	107 (100)	1 (100)	0.62	1.12 [0.72—1.78]	1.12 [0.71—1.79]
Breathing difficulties	97 (80.8)	11 (91.7)	85 (79.4)	1 (100)	0.004	2.01 [1.26—3.33]	2.02 [1.27—3.37]
Rhinitis	6 (5.0)	0 (0,0)	5 (4.7)	0 (0.0)	0.96	0.98 [0.36—2.24]	0.95 [0.35—2.19]
Vomiting	2 (1.2)	0 (0,0)	2 (1.9)	0 (0.0)	0.51	0.61 [0.09–2.16]	0.61 [0.09–2.16]
LRT complications, n (%)							
Pneumonia	37 (30.8)	3 (25.0)	33 (30.8)	1 (100)	< 2.2e^−16^	–	–
Bronchiolitis	69 (57.5)	8 (66.7)	61 (57.0)	0 (0.0)	< 2.2e^−16^	–	–
Acute bronchitis	8 (6.7)	1 (8.3)	7 (6.5)	0 (0.0)	1.73e^−09^	–	–
Asthma exacerbation	11 (9.2)	1 (8.3)	10 (9.3)	0 (0.0)	4.83e^−13^	–	–
Prescribed treatment, n (%)							
Antibiotics	78 (65.0)	10 (83.3)	67 (62.6)	1 (100)	< 2.2e^−16^	–	–
Bronchodilators	40 (33.3)	3 (25.0)	37 (34.6)	0 (0.0)	< 2.2e^−16^	–	–

*n* number of patients, *LRT* lower respiratory tract, *RSV* respiratory syncytial virus.

### Characteristics of inpatients infected with RSV

Demographic characteristics and clinical parameters of RSV-associated SARI patients are presented in Table 1. Among the 120 RSV-positive patients, 55 were male and 65 were female. Their age ranged from 1 month to 31 years, with mean and median ages of 10 months (0.84 year) and 2.4 months (0.2 year) (interquartile range [IQR]: 0.59 year) respectively. With regards to the distribution by age group, we noted that infants aged ≤ 11 months were the most infected group with a detection rate of 83.3% (100/120). RSV infection rapidly dropped after the age of 1 year, with detection rates decreasing from 9.2% in patients aged 1–2 years old (11/120) to 0% in patients aged 11–15 years old (0/120). Only one case of RSV (0.8%) was encountered in patients > 15 years old. At the time of admission, besides cough (75.8%) and fever (43.3%) which were part of case-definition, we found that breathing difficulties (adjusted OR 2.02; 95% CI 1.27–3.37) were more commonly reported among RSV-associated SARI patients (*P-value* = 0.004). Taking into account the clinical diagnosis, more than half of all inpatients infected with RSV had bronchiolitis (57.5%), whereas pneumonia was diagnosed in 37 patients (30.8%). Asthma exacerbation and acute bronchitis were diagnosed in 11 (9.2%) and 8 (6.7%) RSV-associated SARI patients respectively. Antibiotics and bronchodilators were administrated to 65.0% (78/120) and 33.3% (40/120) of patients, respectively (Table 1).

### Co-infection of RSV with other respiratory pathogens

Co-infections among the 120 RSV-positive cases with other community-acquired respiratory pathogens were encountered in 56 (46.7%) patients. Mixed infections with respiratory bacteria were the most commonly observed in this study, with *Haemophilus Influenzae* (17.5%) and *Streptococcus Pneumoniae* (14.2%) being the most co-detected pathogens. Co-infection with *Klebsiella Pneumoniae* was observed in 8 patients (6.7%), *Moraxella Pneumoniae* in 5 patients (4.2%), *Staphylococcus aureus* in 3 patients (2.5%) and *Bordetella Pertussis* (bacterium responsible for whooping cough) in a single patient (0.8%). The most common co-infecting viruses were rhinovirus accounting for 10 cases (8.3%), followed by SARS-CoV2 with 5 cases (4.2%), and HCoV-OC43 with 3 cases (2.5%). RSV/HMPV co-infections were detected in 2 RSV-associated SARI patients (1.7%) (Table 2).

**Table 2:** Co-infection of RSV with other respiratory pathogens.

Pathogens	RSV A	RSV B	Untyped RSV	Overall
	(n = 12)	(n = 107)	(n = 1)	(n = 120)
Viruses, n (%)				
SARS-CoV2	0 (0.0)	5 (4.7)	0 (0.0)	5 (4.2)
Adenovirus	0 (0.0)	2 (1.9)	0 (0.0)	2 (1.7)
HCoV-OC43	0 (0.0)	3 (2.8)	0 (0.0)	3 (2.5)
Enterovirus	0 (0.0)	1 (0.9)	0 (0.0)	1 (0.8)
HMPV	0 (0.0)	2 (1.9)	0 (0.0)	2 (1.7)
Parainfluenza virus	0 (0.0)	2 (1.9)	0 (0.0)	2 (1.7)
Rhinovirus	2 (16.7)	8 (7.5)	0 (0.0)	10 (8.3)
Bacteria, n (%)				
*Haemophilus influenza*	2 (16.7)	19 (17.7)	0 (0.0)	21 (17.5)
*Klebsiella pneumoniae*	0 (0.0)	7 (6.5)	1 (100)	8 (6.7)
*Bordetella Pertussis*	0 (0.0)	1 (0.9)	0 (0.0)	1 (0.8)
*Staphylococcus aureus*	1 (8.3)	1 (0.9)	1 (100)	3 (2.5)
*Moraxella pneumoniae*	2 (16.7)	3 (2.8)	0 (0.0)	5 (4.2)
*Streptococcus pneumoniae*	1 (8.3)	15 (14.0)	1 (100)	17 (14.2)

*SARS-CoV2* severe acute respiratory syndrome coronavirus 2, *HCoV-OC43* human Coronavirus OC43, *HMPV* human metapneumovirus.

### Phylogenetic analysis of RSV-A and RSV-B sequences

**Figure 2:**
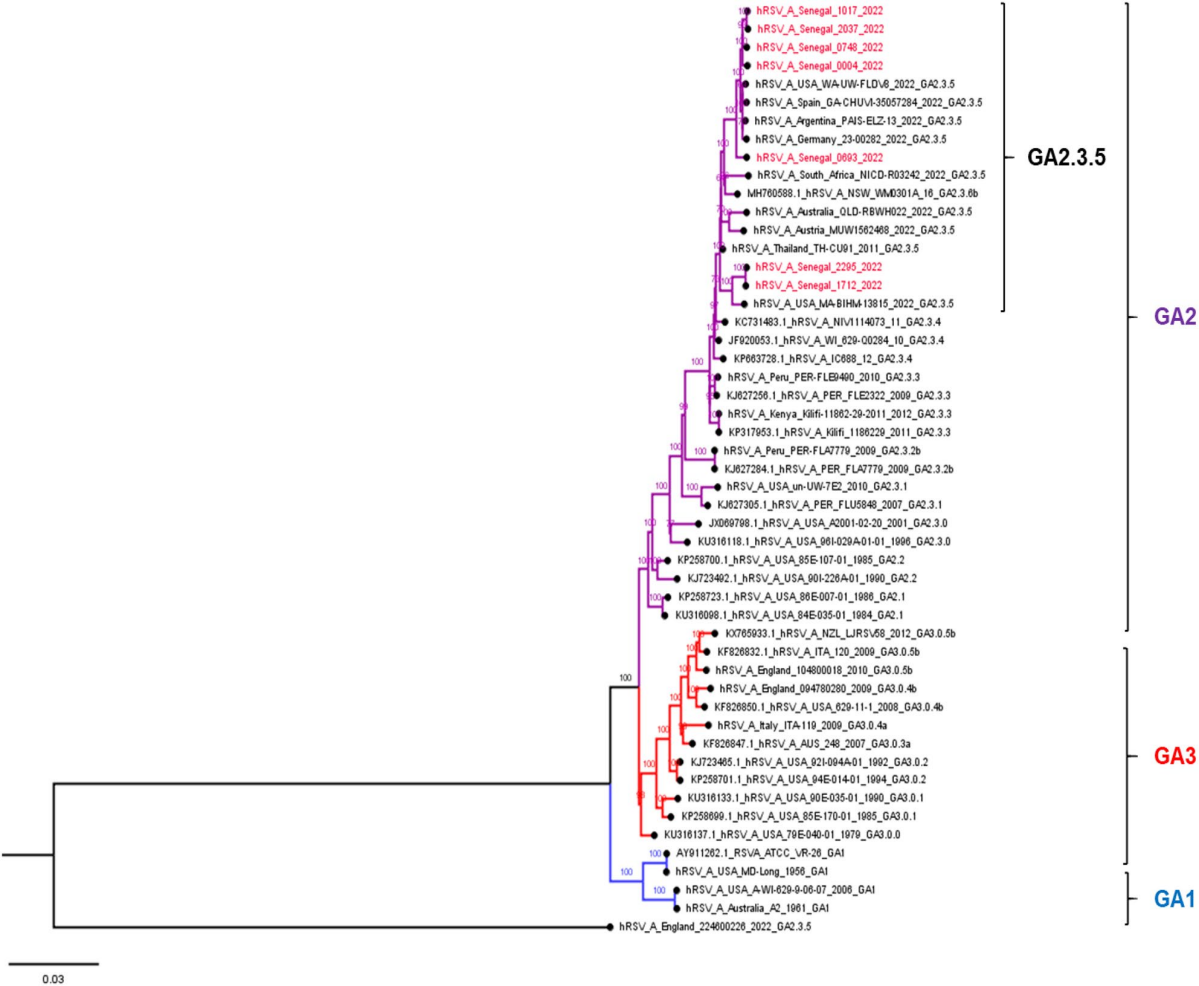
Phylogenetic tree of complete genomes of RSV-A subtype circulating among hospitalized patients in Senegal (January–December 2022). The tree was constructed using the maximum likelihood (ML) method using the IQ-TREE software version 1.6.12^18^ and visualized using the Figtree software version 1.4.4. The statistical significance was tested by 1000 bootstrapping replicates, and the software was responsible for defining the correct model used. Sequences from Senegal are highlighted in red color. The scale bar represents the number of nucleotide substitutions per site.

**Figure 3:**
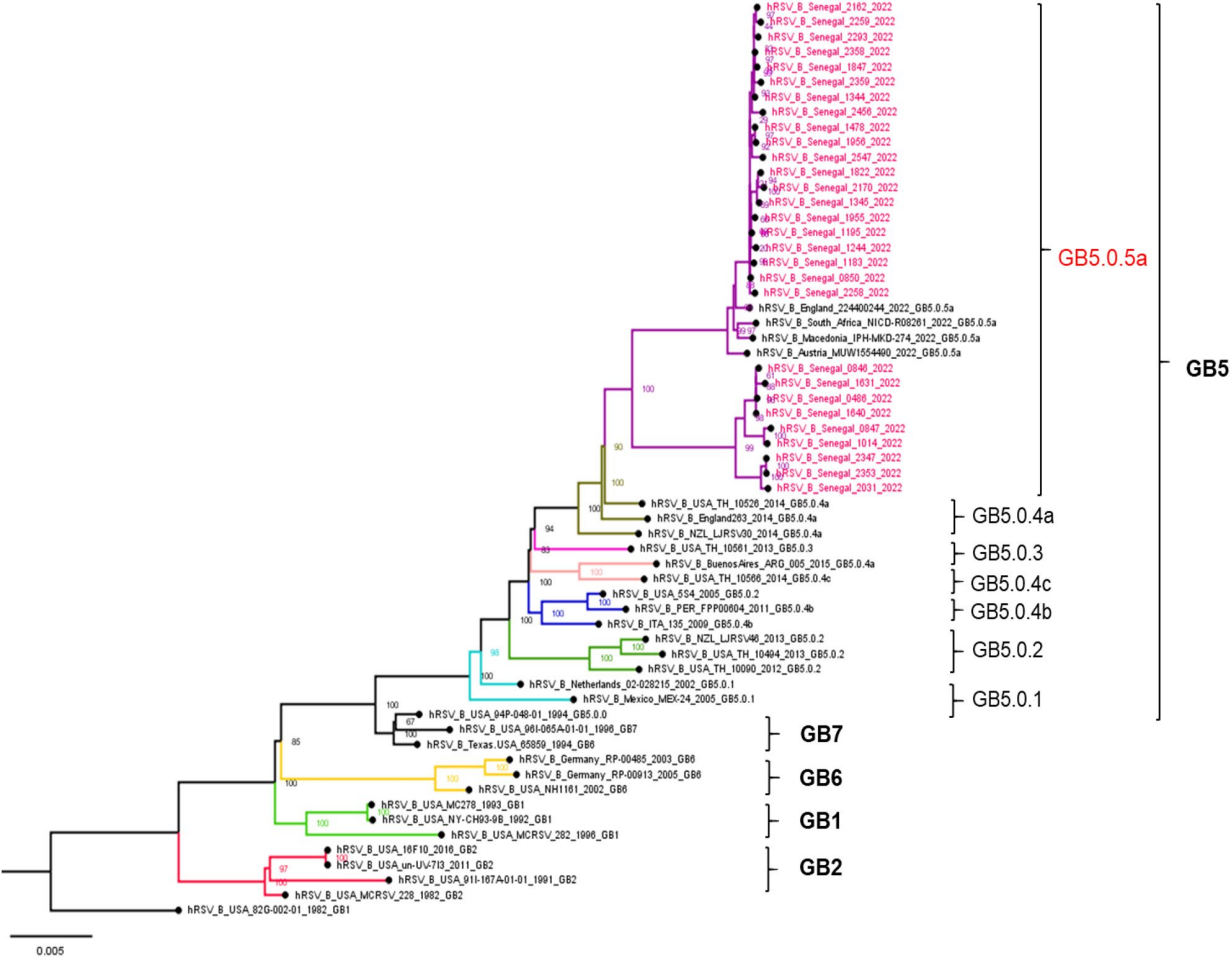
Phylogenetic tree of complete genomes of RSV-B subtype circulating among hospitalized patients in Senegal (January-December 2022). The tree was constructed using the maximum likelihood (ML) method using the IQ-TREE software version 1.6.12^18^ and visualized using the Figtree software version 1.4.4. The statistical significance was tested by 1000 bootstrapping replicates, and the software was responsible for defining the correct model used. Sequences from Senegal are highlighted in red color. The scale bar represents the number of nucleotide substitutions per site.

Overall, 7 RSV-A and 29 RSV-B whole genomes were successfully obtained in this study. The Nextclade online tool (https://clades.nextstrain.org/, accessed 2 August 2023) were initially used for genotype predictions according to Goya et al. 2019^28^. Genotyping of the G gene found that all RSV-A sequences were GA2.3.5 lineage (clade A23), and all RSV-B sequences were GB5.05a lineage (clade B6). We undertook a more detailed phylogenetic analysis of complete genomes with historical and recent RSV sequences in public databases (Genebank and GISAID) up to December 2022 to ascertain more precisely the genetic relationships among RSV strains generated as part of this study and their putative closest ancestors. Phylogenetic trees showed that Senegalese strains were closely related to RSV strains circulating globally in the same period. All RSV-A sequences obtained in this study belonged to the GA2.3.5 genotype (ON1 strains) (Fig. 2), and all RSV-B sequences were classified as genotype GB5.0.5a (BA9 strains) (Fig. 3).

### Genetic analysis of deduced amino acid sequences of RSV strains from Senegal

**Figure 4:**
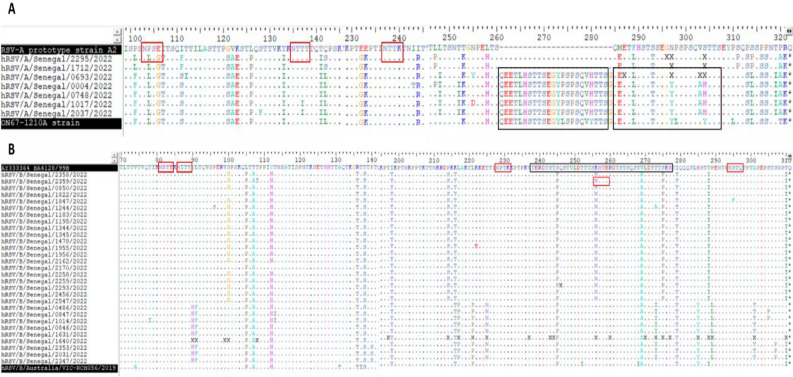
Deduced amino acid alignment and mutations in the second hypervariable region of the G protein of RSV-A (**A**) and RSV-B (**B**) strains from Senegal compared with prototype strain A2 (GenBank accession number M11486) and ON1 reference strain (GenBank accession number JN257693) for RSV-A, BA4128/99B strain (GenBank accession number AY333364) for RSV-B. The two copies of 23 amino acid regions in the RSV-A strains (**A**) and 20 amino acid regions in the RSV-B strains (**B**) are framed black, whereas potential N-glycosylation sites (NXT, where X is not proline) are indicated by red boxed areas. Identical amino acids are indicated by dot and asterisks represent stop codons.

Deduced amino acid sequences of the G protein of RSV-A and RSV-B strains from Senegal were aligned and compared with prototype strain A2 (GenBank accession number M11486) and ON1 reference strain (GenBank accession number JN257693) for RSV‐A and BA4128/99B strain (GenBank accession number AY333364) for RSV-B. For Senegalese RSV-A strains, the 72 nucleotides duplication in the C-terminal end of the G gene, which is characteristic to ON1 strains^18^ was observed in five sequences, leading to an insertion of 24 amino acid (QEETLHSTTSEGYLSPSQVYTTSG) of which 23 are duplications of amino acid spanning positions 261–283 (Fig. 4A). Analysis of all viral genes showed several amino acid substitutions compared to hRSV/A/England/397/2017 (EPI_ISL_412866). All RSV-A samples from Senegal shared mutations P71L, H90Y, I134K, G224E and S243I in the G attachment glycoprotein, V352A in the N protein, L55P in the P protein and S176P in the M2-1 protein. With regards to the fusion glycoprotein F, mutations A103T and T122A which is believed to remove a potential N-glycosylation site at amino acid position 120 (the motif at positions 120–122 changed from NNT (glyco) to NNA (no glyco) were observed only in strains with the 24 amino acid insertion. Using the NetNGlyc 1.0 server, all RSV-A sequences were predicted to have three putative N-glycosylation sites at amino acid positions 103–106 (NLSG), 135–138 (NTTT) and 237–240 (NTTK) such as the ON1 reference strain (JN257693), with the exception of two sequences (hRSV/A/Senegal/1017/2022 and hRSV/A/Senegal/2037/2022) with the substitution T135I leading to the loss of one N-glycosylation site. When compared to the RSV-A prototype strain A2, only one of the three potential N-glycosylation sites predicted in this study remains conserved between all Senegalese RSV-A isolates (amino acid 237 in RSV-A prototype strain A2).

With regards to RSV-B strains, the presence of 60 nucleotides duplication in the 2nd HVR of the G gene, which is a characteristic of the BA genotypes^19^, was found in all sequences, resulting in a duplication of 20 amino acids (TERDTSTSQSTVLDTTTSKH) spanning positions 238–257 (Fig. 4B). In comparison with the hRSV/B/Australia/VIC-RCH056/2019 reference strain (EPI_ISL_1653999) using the GISAID RSVsurver, several mutations were found in most of the viral genes, including the G gene where several amino acid substitutions were found, of which A74V, T131A, I137T, I252T and I268T were present in all RSV-B sequences obtained in this study. All RSV-B strains shared the V97I mutation in the N protein and T1987I in the L protein. Substitutions in the fusion glycoprotein, such as R191K, M206I, R209Q, P312H, S190N, S211N and S389P were identified in most RSV-B sequences from Senegal. When compared with the BA4128/99B strain, S245P and V269A amino acid substitutions in the duplicated 20 amino acid region were observed in all RSV-B strains. The analysis of potential N-glycosylation sites revealed four conserved N-linked glycosylation sites at positions 81–84 (NHTE), 86–89 (NITT), 228–231 (NPTK) and 294–297 (NSTQ) in the 2nd HVR of the G gene of all RSV-B strains in this study in comparison with the BA4128/99B prototype reference strain. The amino acid substitution T310I resulted in a loss of a potential N‐glycosylation site in most of Senegalese strains (25 out of 29), whereas 20 RSV-B strains acquired a putative N‐glycosylation site due to the mutation K256N located in the duplicated 20 amino acid region. Furthermore, amino acid substitution N6S observed in one sample (hRSV/B/Senegal/2031/2022) resulted in the acquisition of a potential N-linked glycosylation.

## Discussion

Before the COVID-19 pandemic, respiratory syncytial virus (RSV) usually circulated primarily in the second half of each year, between June and September in Senegal, which coincide with the period of the rainy season^29^. However, the activity of this virus was disrupted during the COVID-19 pandemic, which circulated at historically low levels due to implementation of public health measures to prevent the spread of SARS-CoV-2 and mitigate the impact of the pandemic. In 2022, after the alleviation of COVID-19 measures, Senegal, like several other countries^26,30–32^, noticed an unprecedented number of RSV detections with a temporal shift (peak of detection between September and October) and increased numbers of pediatric inpatients in the different hospital sentinel sites of the 4S network. The reasons for this atypical resurgence of RSV cases are unclear, but it may be due to a deficit in RSV immunity due to prolonged lack of viral exposure^33^. Furthermore, this resurgence can be explained by specific changes in the circulating genotypes and/or the emergence of a novel strains with increased transmission or pathogenicity^26^. Therefore, to understand whether the increased number of RSV cases seen in the post-pandemic period, was due to specific changes in the circulating genotypes or to the emergence of novel strains, and whether they cause severe illness, we investigated the epidemiology and the genetic characteristics of RSV circulating among hospitalized patients with SARI in Senegal from January to December 2022. Among the 748 samples from the hospital-based surveillance screened as part of this study, 120 were positive for RSV, representing a detection rate of 16.04%. This prevalence is higher than the rate of 11.4% reported in a previous study in Senegal in Influenza-like Illness (ILI) outpatients after four consecutive years of surveillance (2012– 2015)^29^. However, many other similar studies reported higher detection rates of RSV infection, including Thailand^34^ with 27.2%, Germany^35^ with 42.8%, Italy^36^ with 40.6% and Bulgaria^37^ with 26.2%. On the other hand, and in accordance with our current findings, a report in China indicated an overall positivity rate of 16.0% (95% CI = 12.9, 19.6%) among children^38^. The possible reasons for the difference in RSV infection rates could be reflecting the true burden in each region, or it is possibly attributed to the use of different diagnostic methods, study populations (outpatients or hospitalized patients), the sampling period, environmental factors and even the duration of the study. As reported in various countries^26,30,39^, the 2022 surge in Senegal was mainly caused by the RSV-B subtype, with 89.2% of the overall RSV positive cases. Unlike our results, several authors reported RSV-A as the dominant subtype^35,40^. Our study found no mixed infection of RSV-A/RSV-B, despite the reporting of 3.4% of RSV-A/B coinfection in ILI patients in a previous study in Senegal by Fall et al.^29^ or the 2% reported by Hall et al.^41^ in a population-based surveillance of acute respiratory infections among children under 5 years of age in three U.S. counties. However, our findings regarding *Haemophilus Influenzae* and *Streptococcus Pneumoniae* as the most frequently co-detected bacteria with RSV were similar to those of Lin et al^42^. According to some studies, coinfections can exacerbate the severity of RSV disease while others found no conclusive evidence for a link between the presence of coinfection and the disease severity^43,44^. Therefore, further studies will be needed to elucidate the relationships between RSV co-infection and disease severity.

Although this epidemic is atypical, it seems to have a typical age-related RSV risk pattern, with infants aged ≤ 11 months being the most affected groups (83.3% of the overall RSV positive cases) and the risk of RSV infection decreases with increasing age (9.2% in patients aged 1–2 years old and 0% in patients aged 11–15 years old) due to the development of immunity after repeated infections^37^. This reinforces the idea that the implementation of any prophylaxis (vaccine, monoclonal antibodies) should target children under 1 year. Consistent with our findings, several studies have reported this sensitivity of infants, especially in the first year life to RSV infection^35,45–47^. The relative immaturity of the immune system for most RSV genotypes in early childhood may explain this high prevalence of RSV infection in infant^30^. In line with several reports^37,40^, the majority of RSV-associated patients in this study presented with bronchiolitis (57.5%) followed by pneumonia (30.8%). Indeed, bronchiolitis and pneumonia are among the most important RSV-related concerns, as RSV can directly cause viral pneumonia or patients may be additionally co-infected with bacteria as shown in this study, and both can often lead to hospitalization and severe disease course, increasing the risk of ICU transfer, especially in at-risk groups^42,46,48,49^.

Phylogenetic analysis of all available complete genomes of RSV strains infecting patients hospitalized with SARI in Dakar, revealed an exclusive co-circulation of two genetic lineages, GA2.3.5 for RSV-A and GB5.0.5a for RSV-B within the population; however, GB5.0.5a strains dominated over GA2.3.5 during the entire study period. A similar genotypic composition has been observed in several other countries around the world^26,47,50^. These genotypes driving the 2022 RSV outbreak in Senegal had been circulating within the Senegalese population in pre-pandemic years^14^, suggesting that there are no specific changes in RSV since the COVID-19 pandemic began that would account for increased viral spread.

In agreement with previous findings around the world^15,37,40^, the genetic analysis of deduced amino acid indicated that Senegalese strains possessed several amino acid substitutions, particularly in the second hyper variable region of the G protein gene. Amino acid mutations, P71L, H90Y, I134K, G224E, S243I in RSV-A and A74V, T131A, I137T, I252T, I268T in RSV-B were detected in all sequences compared with reference strains hRSV/A/England/397/2017 and hRSV/B/Australia/VIC-RCH056/2019 respectively, which confirms the high genetic variability of the RSV G gene. Unlike the attachment G gene, few key amino acid changes were found in the other remaining genes for both subtypes, including V352A in the N protein, L55P in the P protein and S176P in the M2-1 protein of all RSV-A strains. Similar to the findings of Goya et al.^50^, the amino acid constellation A103T and T122A in the RSV-A fusion protein was encountered only in sequences with the 72 nucleotide duplication in the C-terminal end of the G gene. With regards to RSV-B strains, several mutations were found in most sequences, such as R191K, M206I, R209Q, P312H, S190N, S211N and S389P in the fusion glycoprotein F, V97I in the N protein and T1987I in the L protein. The virus evasion from host immune response recognition may be mediated by the presence of N- and O-linked glycans on the G protein. It has been established that N and O linked glycosylation in G protein can alter the attachment and antigenicity properties of RSV^16,51^. Unlike the findings of Chen et al.^51^ in China, three putative N-glycosylation sites at amino acid positions 103, 135, 237 were predicted among RSV-A strains, while four conserved N-linked glycosylation sites at positions 81, 86, 231 and 294 were identified in RSV-B strains.

However, we pointed out some limitations in our study. The major limitation of the present study is the fact that only hospitalized patients in Dakar were included, and thus our data may not represent the national RSV burden. Therefore, it would be important to extend the hospital-based surveillance in other regions to firmly establish the burden associated with RSV in Senegal, and clear correlations between disease severity and genotypes.

In summary, this hospital-based surveillance revealed that the abnormal increase in RSV‐positive rates after the alleviation of the COVID-19 measures in Senegal, was mainly due to RSV-B subtype. Globally, we observed a shift in the circulation pattern of RSV (systematic increase in infections between August and November), which usually circulates during the rainy season between June through September each year. Phylogenetic analyses highlighted that all RSV-B strains belonged to GB5.0.5a (BA9 lineage) genotype very similar to that previously circulating, suggesting that the increased number of RSV cases, especially in infants aged ≤ 11 months is likely because of diminished protective immunity in the population from low RSV exposure, a consequence of pandemic mitigation measures. Therefore, a deep molecular surveillance of respiratory‐associated viruses should be established under the COVID‐19 strategy to avoid unexpected outbreaks of other diseases.

## Material and methods

### Study design, sample and data collection

**Figure 5:**
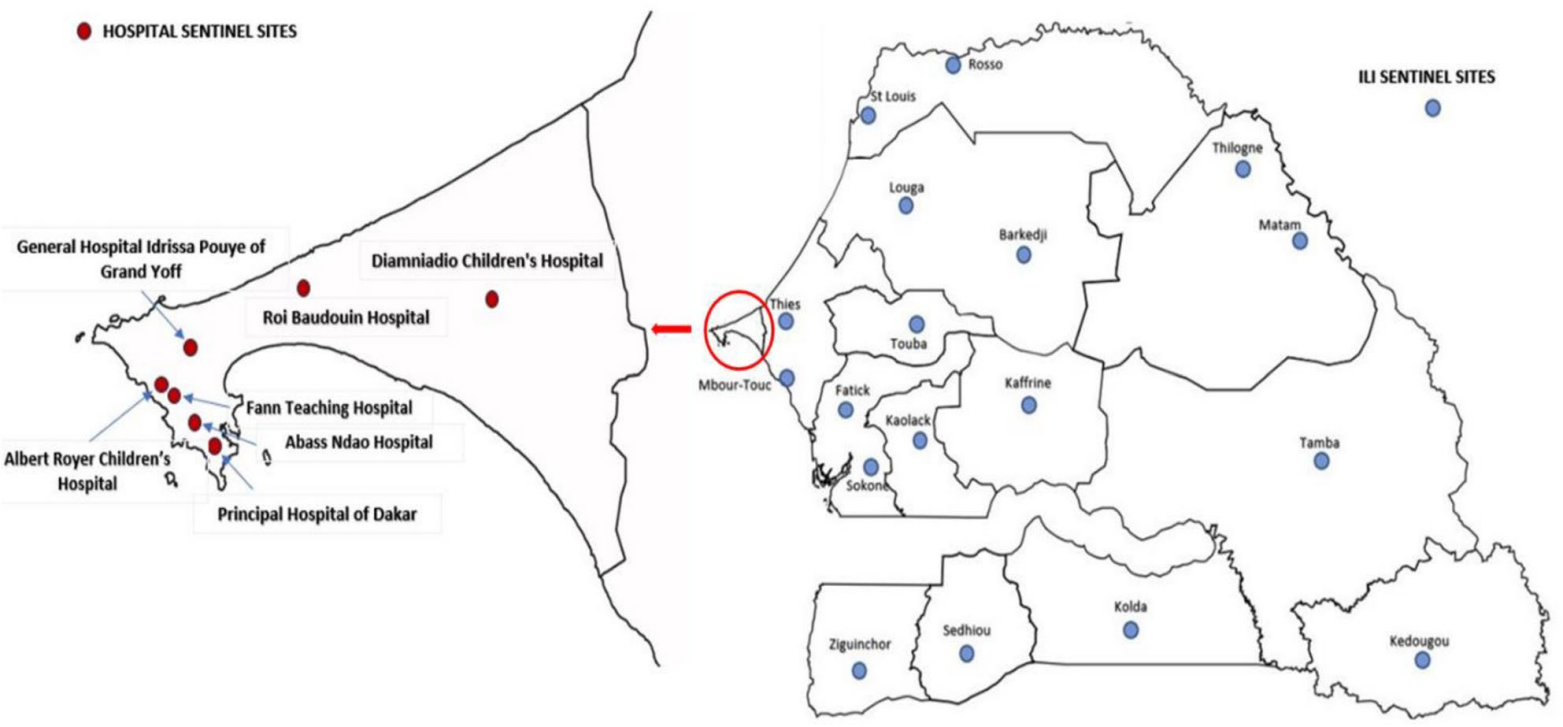
Map of Senegal showing the different sentinel sites of the 4S network. Hospital sentinel sites, all located in the capital Dakar are represented by red dots, while blue dots represent the ILI community sentinel sites.

This study was conducted from January to December 2022 and samples were collected as part of the routine hospital-based surveillance of SARI in Senegal piloted by the National Influenza Centre (NIC) hosted at the Institut Pasteur de Dakar (IPD). A standardized SARI case definition according to the WHO was followed, which defines SARI as “an acute respiratory infection with history of fever or measured fever of ≥ 38 °C, and cough with onset within the last ten days and requires hospitalization”^52^. Participants were enrolled in seven sentinel hospitals (all located in the capital city Dakar) for the surveillance of SARI: Albert Royer Children's Hospital, Diamniadio Children's Hospital, General Hospital Idrissa Pouye of Grand Yoff, Fann Teaching Hospital, Principal Hospital of Dakar, Abass Ndao Hospital and Roi Baudouin Hospital (Fig. 5). Upon admission, nasopharyngeal and/or oropharyngeal swabs were collected per patient by qualified medical personnel. The swabs were inserted in a universal viral transport medium (Becton Dickinson and company, Milano, Italy) and promptly transported at a controlled temperature (4–8 °C) to the laboratory for the screening of respiratory pathogens, including RSV within the 24 h following samples collection. In addition, for each identified patient admitted at the selected hospitals, demographic, clinical, and epidemiological information for all SARI cases were recorded in a case-based surveillance form by trained nurses. Upon receipt in the laboratory, samples were tested using a multiplex real-time reverse transcription polymerase chain reaction assay and left-over clinical specimens were stored at − 80 °C for biobanking and additional analyses (e.g., NGS sequencing).

### Nucleic acid extraction and screening of respiratory pathogens

Total nucleic acid was extracted from 200 μl of swab suspension in Viral Transport Medium (VTM) using the QIAamp Viral RNA kit (QIAGEN, Valencia, CA, USA), according to the manufacturer’s instructions. The extracted nucleic acid was eluted in a final volume of 60 µl and immediately used for the routine testing of respiratory pathogens. Extracted RNA from all samples was tested for RSV, along with other respiratory pathogens (Virus and bacteria) by multiplex real time reverse transcription polymerase chain reaction (rRT-PCR) system, using the Allplex™ Respiratory Full Panel Assay (Seegene, Seoul, Republic of Korea). This multiplex one-step real-time RT-PCR assay that composed of 4 different panels enables the simultaneous detection and differentiation of 26 causative pathogens in respiratory tract infections including 16 viruses, 3 Flu A subtypes and 7 bacteria. Respiratory syncytial virus A (RSV-A) and Respiratory syncytial virus B (RSV-B) along with the 3 Flu A subtypes (A/H1pdm09, A/H3 and A/H1) were detected with the Panel 1. Briefly, the total reaction volume for each real time PCR was 25 μl, consisting of 5 μl Nuclease-free water, 5 μl of 5XRP MOM (MuDT Oligo Mix), 5 μl of 5X Buffer (Buffer containing dNTPs), 2 μl of Enzyme Mix and 8 μl of each extracted or controls nucleic acid under the following cycling conditions: reverse transcription step of 20 min at 50 °C, initial denaturation step of 15 min at 95 °C, followed by 45 PCR cycles of 10 s at 95 °C, 1 min at 60 °C and 10 s at 72 °C. Fluorescence is detected at 60 °C and 72 °C. The real-time RT-PCR was done on the CFX96™ (Bio-Rad, California, USA) platform, and subsequently interpreted by Seegene’s Viewer software. The latter considers respiratory targets that generate adequate, exponential fluorescence curve with Ct-values or cycle threshold value (it represents the number of cycles required for the fluorescent signal to cross the threshold. It is inversely proportional to the amount of target nucleic acid in the sample), below 42 cycles as positive.

### Next generation sequencing of RSV positive samples

For RSV whole genome sequencing, stored positive samples with a relatively high viral load (Ct-value < 30) were selected and transmitted to the IPD sequencing platform to perform hybridization capture-based, metagenomic next-generation sequencing using the Twist Respiratory Virus Research Panel (103067; Twist Biosciences, San Francisco, CA), as previously described^53^. The panel is a culture-free, hybrid capture enrichment workflow embedded as a sub analysis panel in One Codex analysis platform and is capable of detecting 29 common human respiratory viruses simultaneously^54^. To generate the consensus genomes of RSV in all samples, raw reads from Illumina sequencing in FASTQ format were assembled by de novo assembling using CZ ID (https://czid.org/, accessed on June 21, 2023), a cloud based open-source bioinformatics pipeline for metagenomic sequencing data.

### Sequence alignments and phylogenetic analysis

Phylogenetic analyses were carried out by adding sequences from other countries strains downloaded from GenBank (https://www.ncbi.nlm.nih.go, accessed on 21 June 2023) and GISAID (https://www.gisaid.org, accessed on 21 June 2023) databases, as well as adding reference sequences of known genotypes. Sequences alignments were performed by using MAFFT software implementing the FFT-NS-2 algorithm^55^. The alignment was manually inspected in Bioedit v7.1.3.0^56^ to ensure accuracy and that identical sequences were removed. The alignment was used to construct phylogenetic trees by means of the maximum likelihood method by using IQ-TREE v.2.1 with ultrafast bootstrap and SH-aLRT (1000 replicates each) to assess the phylogenetic clades statistical support^57^. Trees were visualized using FigTree version 1.4.4 (http://tree.bio.ed.ac.uk/software/figtree/, accessed on 21 June 2023). Based on the clustering in the phylogenetic tree and supported by bootstrap values, sequences generated in this study are categorized into specific genotypes. Robustness of trees topology was accessed with 1000 replicates, and bootstrap values ≥ 70% were considered significant.

### Deduced amino acid sequence analysis

Deduced amino acid sequences were translated with the standard genetic code using the MEGA7 software^58^. The GISAID RSVsurver (https://www.epicov.org/epi3/frontend#1c1efe, accessed on 04 August 2023) was used to identify key amino acid mutations. Potential N-glycosylation sites in amino acid sequences of both RSV-A and RSV-B G proteins were predicted with threshold of 0.5 by using NetNGlyc 1.0 webserver (http://www.cbs.dtu.dk/services/NetNGlyc, accessed on 04 August 2023).

### Statistical analysis

SARI case-based data were entered into an EpiInfo database, merged with laboratory results, and subsequently analyzed using *R* statistical software (R.3.0.1 version). Chi-square (χ^2^) and Fisher’s exact tests were used to support the comparisons of the categorical data, where a *p*-value < 0.05 was considered statistically significant. The proportions were reported with 95% confidence intervals (CIs).

### Ethical considerations

This study was conducted as part of the hospital-based surveillance of severe acute respiratory infection of the 4S (Syndromic Sentinel Surveillance in Senegal) Network, which has the approval from the Senegalese National Ethical Committee of the Ministry of Health as being less than minimal risk research. All methods were carried out in accordance with relevant guidelines and regulations, and verbal informed consent was obtained from all subjects and/or their legal guardian(s). The protocol and oral consent were determined as routine surveillance activity, and therefore non-research by the Senegalese National Ethics committee and the steering committee for 4S network, an entity representing MoH, Institut Pasteur Dakar, WHO and Clinicians in compliance with all applicable National regulations governing the protection of human subjects. Data were collected in an objective of surveillance and are anonymous. The information provided to participants was an informal description of the study. Respiratory specimens were collected, only after informed consent was granted, verbally, to local health care workers by the patients or parents in the case of minors. Patients could refuse to participate; no specimen will be taken. For the surveillance activities, written consent is judged not necessary by the Senegalese national ethics committee, which has also previously approved the work of the National Influenza Center. Collections of non-sensitive data or an observation from normal care in which participants remain anonymous do not require ethics committee review. The patients included in this study were of all ages and consulted the Hospital sites due to acute respiratory syndromes; the patients, or parents in the case of minors, accept the tests for respiratory viruses largely because they are free and safe. The data is available in real-time to the Epidemiology Department at the Senegalese Ministry of Health and Prevention to support the appropriate public health action.

## Data Availability

All data generated during this study are contained within this manuscript. All RSV sequences generated in this study have been deposited in the GISAID database (https://gisaid.org/) under accession ID EPI_ISL_18228265, EPI_ISL_18228290, EPI_ISL_18228291, EPI_ISL_18228292, EPI_ISL_18228293, EPI_ISL_18228294, EPI_ISL_18228295, EPI_ISL_18228533, EPI_ISL_18228536, EPI_ISL_18228537, EPI_ISL_18228539, EPI_ISL_18228540, EPI_ISL_18228541, EPI_ISL_18228544, EPI_ISL_18228548, EPI_ISL_18228618, EPI_ISL_18228653, EPI_ISL_18228657, EPI_ISL_18228658, EPI_ISL_18228659, EPI_ISL_18228660, EPI_ISL_18228661, EPI_ISL_18228662, EPI_ISL_18228663, EPI_ISL_18228703, EPI_ISL_18228704, EPI_ISL_18228705, EPI_ISL_18228706, EPI_ISL_18228707, EPI_ISL_18228708, EPI_ISL_18228709, EPI_ISL_18228811, EPI_ISL_18229075, EPI_ISL_18229381, EPI_ISL_18229635, EPI_ISL_18229703.
